# Preeclampsia Is a Biomarker for Vascular Disease in Both Mother and Child: The Need for a Medical Alert System

**DOI:** 10.1155/2013/953150

**Published:** 2013-04-16

**Authors:** Julie Hakim, Mary K. Senterman, Antoine M. Hakim

**Affiliations:** ^1^Department of Obstetrics and Gynecology, The Ottawa Hospital and the University of Ottawa, General Campus, 501 Smyth Road, Ottawa, ON, Canada K1H 8L6; ^2^Departments of Pathology & Laboratory Medicine and Obstetrics & Gynecology, The Ottawa Hospital and the University of Ottawa, General Campus, 501 Smyth Road, Ottawa, ON, Canada K1H 8L6; ^3^Division of Neurology, The Ottawa Hospital and the University of Ottawa, Ottawa, ON, Canada K1H 8M5; ^4^Neuroscience Research, Ottawa Hospital Research Institute, Ottawa, ON, Canada K1Y 4E9; ^5^Canadian Stroke Network, Ottawa, ON, Canada K1G 5Z3; ^6^Brain and Mind Research Institute, University of Ottawa, 2413-451 Smyth Road, Ottawa, ON, Canada K1H 8M5

## Abstract

This paper reviews the literature pertaining to the impact of preeclampsia not only on the mother but particularly on the children. The review points to the higher blood pressure in children born to preeclamptic mothers compared to controls, their increased tendency to suffer strokes, the reduction in their cognitive ability, and their vulnerability to depression. Mechanisms that may induce these changes are emphasized, particularly the placental vascular insufficiency and the resulting hypoxic and proinflammatory environments in which the fetus develops. The hypothesis proposed is that these changes in the fetal-placental environment result in epigenetic programming of the child towards a higher propensity for vascular disease. The review's main recommendation is that, within ethical boundaries, the medical records of individuals born to preeclamptic mothers should clearly indicate this event and should be made available to the affected individuals so that preventive measures against vascular complications and lifestyle changes that may mitigate the latter can be instituted.

## 1. Introduction

Pregnancy is a transient condition, but when it is complicated by preeclampsia it has lasting effects on both the mother and the child. This paper describes the short- and long-term consequences of preeclampsia to both the mother and the child, with an emphasis on the children's vulnerability to vascular disease and cognitive impairment, and summarizes the potential pathophysiologic mechanisms at play. It also aims to expose the need to more easily identify individuals whose mothers were preeclamptic so that prevention measures can be instituted to avoid the vascular disease complications. 

The paper is the result of multiple literature searches through PubMed and other search engines spanning the period from 2000 to 2012. Preference was given to meta-analyses and large health surveys. The articles referenced in this paper were selected because they contributed to understanding the broader picture emerging on the importance of preeclampsia as a long-term threat to the health of the mother and the child. 

## 2. Definition and Prevalence of Preeclampsia

The American College of Obstetricians and Gynecologists defines preeclampsia as hypertension with a systolic blood pressure of 140 mm/Hg or higher or a diastolic blood pressure of 90 mm/Hg or higher that occurs after 20 weeks of gestation in a woman with previously normal blood pressure. The hypertension must be combined with proteinuria, defined as urinary excretion of 0.3 g protein or higher in a 24 h urine collection [1]. Increasingly, however, it is appreciated that preeclampsia is a systemic disease that has both short- and-long term consequences to both mother and offspring. As well, there is concern that the incidence and prevalence of preeclampsia are rising in society with the increase in some of the known risk factors for this condition. 

Worldwide, the prevalence of preeclampsia ranges from 3 to 8% of all pregnancies [2]. The prevalence, however, appears to vary by location and by ethnic background. In a large study of almost a million women from New York City, the prevalence of preeclampsia was 3.2%. Linking this event with ethnicity showed that East Indian women had the lowest risk of preeclampsia (1.4%) and Mexican women had the highest risk (5.0%) [3]. The ECLAXIR Study in France showed that African origin was a risk factor for preeclampsia [4].

## 3. Risk Factors and Predispositions for Preeclampsia 

Women of advanced maternal age exhibit more preeclampsia than younger women. A registry-based study from Finland showed that under the age of 35 the rate of preeclampsia was 6.4%, but in women older than 35 the rate climbed to 9.4% [5]. This study additionally identified other associations with preeclampsia, such as a body mass index (BMI) of more than 25 and a higher incidence of maternal diabetes and chronic hypertension. This was confirmed by two recent studies, one that included 220 patients from Saudi Arabia and showed that patients with gestational diabetes mellitus had a significantly higher incidence of preeclampsia as well as preterm delivery [6] and a study of 2,056 pregnancies from Qatar which revealed that the incidence of preeclampsia was 7.3% in women with, and 3.8% in women without, gestational diabetes [7]. In contrast, a large Norwegian study that included more than 200 women giving birth from 1967 to 2008 showed that the rates of preeclampsia increased more over time among younger than older women [8]. Another study from USA showed that gestational hypertension, preeclampsia in a prior pregnancy, a BMI of more than 30, and African American race were all predictive of late postpartum preeclampsia [9], and a study from Taiwan confirmed that elevated BMI increased the risks of gestational diabetes mellitus, preeclampsia, and preterm labour [10]. In teenage deliveries, defined as mothers ≤18 years of age, 8.9% developed preeclampsia, and the strongest associations were with BMI of more than 40 and elevated gestational weight gain [11]. Thus, elevated BMI and gestational diabetes consistently appear as risk factors for preeclampsia in the mother.

## 4. Consequences of Preeclampsia to the Mother

Although preeclampsia is a transient phenomenon, there are both acute and long-term significant consequences to the mother affected by this condition. The condition is associated with significant mortality, and black women were 3.1 times more likely to die with this condition than white women [12]. In mothers who survive, there are a number of short- and long-term consequences to preeclampsia.

### 4.1. Short-Term Maternal Consequences of Preeclampsia

#### 4.1.1. Vascular

Women who have had a pregnancy complicated by preeclampsia have an increased risk of antenatal stroke [13, 14]. This serious complication of pregnancy was associated with a previous history of migraine and gestational diabetes [13]. Some insight into the pathophysiology for increased stroke risk was derived from a study of 40 women with untreated preeclampsia who were compared to 40 matched healthy pregnant women [15]. Those with untreated preeclampsia demonstrated increased cardiac output and cardiac stroke volume as well as increased systemic vascular resistance, which led the authors to conclude that the hypertension associated with untreated preeclampsia is due to increased cardiac output and mild vasoconstriction with reduced diastolic function. This was confirmed by a recent echocardiographic study [16]. Khalil and colleagues, in a screening study, showed that women who develop preeclampsia had higher aortic systolic blood pressure and arterial stiffness, which are apparent from the first trimester of pregnancy [17]. In addition, stroke risk is greater when the pregnancy is associated with acute microalbuminuria in the absence of preexisting kidney disease, and the association persists even after adjustment for established cardiovascular risk factors [18].

#### 4.1.2. Nonvascular

A number of additional clinically relevant nonvascular consequences to the mother may be noted during the preeclampsia phase, confirming that preeclampsia is a syndrome with multiorgan impact. There is a deterioration of maternal renal function with the possibility of a rise in serum creatinine to more than 0.9 g/L, liver involvement with elevated liver enzymes, pulmonary edema (particularly in cases of severe preeclampsia), hematological disorders including thrombocytopenia, hemolysis and disseminated intravascular coagulation, neurological involvement with visual disturbances, severe headaches and hyperreflexia, and intrauterine growth restriction [19, 20]. As well, women with preeclampsia in the 3rd trimester showed significantly higher levels of serum procalcitonin, C-reactive protein (CRP), and plasma D-dimer levels, and these hematological indices were significantly higher in patients with severe as compared to mild preeclampsia [21]. The rate of very early preterm delivery (less than 32 weeks of pregnancy) was 21.2% in uncomplicated preeclampsia compared with 37.2% in preeclampsia complicated by prior chronic hypertension [22].

### 4.2. Long-Term Consequences of Preeclampsia to the Mother

Most long-term consequences of preeclampsia in the mother are vascular. A recent paper by Smith and colleagues estimated the 10-year, 30-year, and lifetime cardiovascular disease (CVD) risk following a pregnancy complicated by preeclampsia by comparing CVD events in 118 control women and 99 preeclamptic women [23]. A total of 18.2% of preeclamptic women and 1.7% of control women had a high 10-year risk (OR 13.08; 95% confidence interval (Cl) 3.38 to 85.5), 31.3% of preeclamptic women and 5.1% of control women had a high 30-year risk (OR 8.43; 95% Cl 3.48 to 23.23), and 41.4% of preeclamptic women and 17.8% of control women had a high lifetime risk for CVD (OR 3.25; 95% Cl 1.76 to 6.11). Davis and colleagues state that women who have had a pregnancy complicated by preeclampsia have a 4-fold increased risk of later cardiovascular disease [24].

There is also increased cerebrovascular risk to preeclamptic women later in life. When 73 formerly preeclamptic women were matched for age and time since pregnancy with control women, MRI scans in the formerly preeclamptic women showed significantly more frequent and more severe white matter brain lesions [25]. This increased cerebrovascular risk was measured 18 years after pregnancy in a prospective cohort study of more than 3,000 women. It showed that the calculated 10-year cerebrovascular disease risk in preeclampsia had an odds ratio of 1.31 (95% CI: 1.11, 1.53) compared to women without preeclampsia [26]. The authors suggested that preeclampsia may be a better predictor of future cerebrovascular disease than other pregnancy-associated abnormalities. This is confirmed by a nationwide study from Sweden which reviewed almost one million women. The study showed that the risk of maternal cerebrovascular disease increased with decreasing gestational age. The hazard ratio of cerebrovascular disease ranged from 1.39 (95% CI 1.22–1.53) to 2.57 (95% CI 1.97–3.34) for mothers with small-for-gestational age births [27]. 

Five- to 8-years postpartum, Kvehaugen and colleagues in Norway compared 26 mother and child pairs from pregnancies complicated by preeclampsia with pairs from uncomplicated pregnancies and showed that endothelial function was significantly reduced in both mothers and children after preeclampsia, especially when combined with small-for-gestational age infants. This included measurements of fMS-like tyrosine kinase and CRP which were elevated in the preeclampsia group compared with controls. These changes were also found in the infants [28]. CRP has been correlated with vascular complications and is now accepted and used as a marker for vascular vulnerability [29]. Another study has recently confirmed the relationship between 2nd trimester antiangiogenic proteins and preeclampsia [30].

These studies show conclusively that mothers who were exposed to preeclamptic pregnancies have a higher incidence of cerebrovascular and cardiovascular disease. Although there have been no studies on possible cognitive impairment in women who gave birth associated with preeclampsia, the increased cerebrovascular risk and the presence of white matter lesions on MRI scans have been associated with this outcome in several other studies [31, 32].

## 5. Short- and Long-Term Consequences of Preeclampsia to the Offspring

A number of cardiovascular and other complications have been reported in children born to preeclamptic mothers. 

### 5.1. Cardiovascular, Cerebrovascular, Cognitive, and Psychiatric

A systematic review and meta-analysis on studies reporting traditional cardiovascular risk factors in children exposed to preeclampsia compared to controls showed a 2.39 mm/Hg (95% CI: 1.74–3.05; *P* < 0.0001) higher systolic and a 1.35 mm/Hg higher diastolic blood pressure (95% CI: 0.90–1.80; *P* < 0.0001) during childhood and young adulthood. As well, the BMI of these children was significantly higher, but there was insufficient evidence to identify consistent variation in lipid profile or glucose metabolism [24]. An Australian study, on the other hand, showed that offspring of women who experienced hypertensive disorders of pregnancy had 3.46 mm/Hg greater systolic blood pressure and 3.02 mm/Hg greater diastolic blood pressure at age 21 years [33]. In a large UK cohort, assessed at age 9–12 years after pregnancy, including maternal-offspring pairs, Lawlor and colleagues reported that offspring of women with preeclampsia had higher systolic blood pressure by 2.04 mm/Hg even when the analyses were adjusted for maternal and offspring BMI, sodium intake, and other potential confounders [34]. Although these blood pressure elevations appear modest, it is important to bear in mind that hypertension in children, regardless of etiology, can result in significant end-organ damage [35, 36].

Knowing that hypertension increases the risk for stroke, it is perhaps not surprising that Kajantie and colleagues have reported that preeclampsia is associated with increased risk of stroke in the adult offspring [37]. The crude hazard ratio for all forms of stroke among individuals whose mothers had preeclampsia was 1.9 (1.2 to 3.0; *P* = 0.01), and among people whose mothers had gestational hypertension, it was 1.4 (1.0 to 1.8: *P* = 0.03).

Fugelseth et al. reported that 45 children delivered from pregnancies complicated by preeclampsia had significantly smaller hearts, increased heart rate, and increased late diastolic velocity (A-wave, at mitral valve attachments), supporting the conclusion that the cardiovascular phenotype of offspring of preeclampsia mothers is altered [38].

Age-related change in cognitive ability in the offspring of mothers who were hypertensive during pregnancy was studied by a group in Finland. They showed that men born after pregnancies complicated by hypertensive disorder scored 4.36 points lower on total cognitive ability at 68.5 years (95% CI, 1.17–7.55). They also displayed a greater decline in total cognitive ability [39]. Because this was a study of military forces, women were not included. Recently, a study from Australia confirmed this association. Verbal ability at age 10 years was assessed with the Peabody Picture Vocabulary Test-Revised (PPVT-R) and nonverbal ability with Raven's coloured progressive matrices (CPM). Offspring of pregnancies complicated by maternal hypertension or preeclampsia had a mean PPVT-R score that was 1.83 (*P* = 0.03) point slower than children from normotensive pregnancies [40], and there was no significant association between offspring gender and PPVT-R or CPM scores. The authors conclude that maternal hypertensive diseases of pregnancy are a risk factor for a small reduction in offspring verbal ability. 

Children of preeclamptic mothers also suffer from psychiatric disorders. Another study by Tuovinen and colleagues in Finland showed that female offspring born to pregnancies complicated by hypertension without proteinuria were at 1.19-fold higher risk of mental disorders (CI: 1.01–1.41, *P* = 0.04) and similarly showed significant increases in the risk of mood and anxiety disorders (CI: 1.11–1.88, *P* < 0.01) [41]. In contrast, preeclampsia was associated with a lower risk of any mental disorder in the male offspring. The same group had reported earlier that primiparous pregnancies complicated by preeclampsia were associated with later depressive symptoms in the progeny, regardless of the children's gender [42].

### 5.2. Other Complications of Preeclampsia in the Offspring

Ozkan and colleagues have reported that the incidence of bronchopulmonary dysplasia in preterm infants born to a group of preeclamptic mothers was 38.5%, and was significantly higher than that in those born to normotensive mothers, reported at 19.5% [43]. There is also some evidence to suggest that if the pregnancy is associated with prior chronic hypertension in the mother, then a number of additional structural birth defects may occur [44]. Thus, preeclampsia may have significantly more consequences to children born of mothers with prior cardiovascular disease. 

Interestingly, Wikström and colleagues revealed that there is an intergenerational recurrence of placental dysfunction disorders, such that mothers who were born small-for-gestational age (SGA) suffered disproportionately and significantly from preeclampsia, placental abruption, spontaneous preterm birth and still birth [45]. Compared with parents who had not been born SGA, the risk of preeclampsia was more than 3-fold increased if both parents had been born SGA, whereas if only the mother had been born SGA the corresponding risk was increased by 50%. The rate of small for gestational age infants increased to 50.7% in preeclampsia versus 5% in the controls [22].

## 6. Suspected Mechanisms for Mother-to-Child Transmission

The main pathways by which preeclampsia serves to modify vascular risk would appear to be hypoxia, antiangiogenesis, endothelial dysfunction and immune modifications. These pathways individually, synergistically, or cumulatively appear to alter the epigenetic potential of offspring exposed to a preeclampsia environment *in utero* leading to altered vascular phenotype after birth. 

### 6.1. Reduced Uterine Perfusion and Hypoxia

A key feature of the *in utero* preeclamptic environment is reduced uterine perfusion of the fetus-placental unit leading to hypoxia and placental release of reactive oxygen species (ROS) and cytokines [46]. The progression to a systemic oxidative and inflammatory state arises from the placenta overexpressing antiangiogenic factors which inhibit the normal pregnancy-related angiogenesis and contribute to systemic endothelial dysfunction. Thus, offspring of preeclamptic mothers develop in an environment of placental insufficiency and hypoxia with exposure to circulating inflammatory and antiangiogenic factors. These alter long-term risk for vascular disease, likely by activating mechanisms that decrease transcriptional activity in the endothelium or alter vascular gene expression. Thus, abnormal placentation in early pregnancy is considered the key pathological insult leading to subsequent development of the preeclampsia syndrome [46]. 

In animal studies, mechanical reduction in uterine artery perfusion pressure (RUPP) and blood flow in rats between 14 and 19 days of gestation induces a full preeclampsia like phenotype, including elevation in blood pressure, changes in renal artery flow [47], and dysfunction of endothelium-dependent relaxation [48]. As well, there is subsequent release of endothelial microparticles resulting in the binding of the proangiogenic factors, placental growth factor (PIGF) and vascular endothelial growth factor (VEGF) [46]. Variations in the timing of RUPP (early versus late gestation) have shown that early exposure to perfusion abnormalities appears critical for altering blood pressure programming in the offspring [49]. A review of the advantages and disadvantages of this model was recently published [50].

The early placental insufficiency that persists in preeclampsia leads to an environment of significant hypoxia. Lai and colleagues have shown that mice with normal placentation that are exposed to significant hypoxia, coupled with the deficiency of the interleukin IL-10 from early pregnancy, will develop preeclampsia like symptoms including hypertension, proteinuria, renal pathology, and intrauterine growth restriction [51]. Because the offspring of these pregnancies, complicated by preeclampsia-like symptoms, did not show elevations in blood pressure, it is thought that hypoxia alone may be insufficient to cause offspring alterations in blood pressure but can act as a trigger to what is now referred to as the “preeclampsia cascade” [46].

### 6.2. Endothelial Dysfunction and Reduced Angiogenesis

Along with early placental insufficiency and hypoxia, endothelial dysfunction is a significant pathophysiologic mechanism leading to the development of the preeclampsia syndrome [52]. As well, once established, endothelial function remains impaired in women with previous preeclampsia [53]. The impact of systemic endothelial dysfunction on *in utero* development can be shown in mouse models genetically manipulated to develop systemic inhibition of endothelial nitric oxide synthase (eNOS) [54]. When these mice are bred with wild-type males to produce eNOS-heterozygous offspring, these offspring have higher BP during adulthood than genetically similar offspring from wild-type mothers and eNOS-knockout fathers. This distinction serves to highlight the impact of maternal endothelial dysfunction on *in utero* development of subsequent blood pressure irregularities in the offspring [55].

Directly related to reduced nitric oxide availability and its subsequent endothelial dysfunction are alterations in angiogenic factors. Elevations in circulating antiangiogenic factors are viewed as a key step in the progression to preeclampsia from the initiating event of placental insufficiency. Persistence of angiogenic abnormalities in the offspring are also thought to lead to persistent endothelial dysfunction later in life [47]. In animal models Maynard and colleagues developed a Sprague-Dawley rat model of preeclampsia based on adenovirus vector administration of sFlt-1, a competitive binder of placental growth factor, at days 8-9 of pregnancy [56]. These transfected rats developed hypertension, heavy proteinuria, and glomerular endotheliosis. Lu and colleagues also showed that male offspring of mice transfected with sFlt-1 showed a sustained increase in blood pressure from day 1 of life [57]. Petrozella and colleagues implicate sFlt-1 as well as soluble endoglin (sEng) proteins in human preeclampsia [58]. Released from the placenta in response to diffuse endothelial injury, these factors have significant effects on maternal vasculature. Though the placenta normally produces sFlt-1 and sEng, these factors are produced in higher amounts from hypoxic placentae in pregnancies affected by preeclampsia and interact with PIGF and VEGF in women destined to develop preeclampsia. The authors suggest that the antiangiogenic environment is best reflected by the sFlt1 : PIGF ratio which they feel provides a snapshot of antiangiogenic imbalance [58]. LaMarca and colleagues have recently reported that RUPP is a stimulus for angiotensin II type I receptor during pregnancy and emphasized the important role that stimulating this receptor plays in preeclampsia [59]. 

### 6.3. The Role of Microparticles

To directly assess the level of endothelial damage, quantification of endothelial microparticles (EMPs) may be necessary. These are submicroscopic membranous particles shed from the endothelial cell wall upon disturbance which can be identified in the plasma by fluorescent antibody labelling and quantified with flow cytometry. A higher total number of microparticles were observed in women with severe preeclampsia compared with normotensive pregnant women and nonpregnant women [60]. The endothelial microparticles CD31+/42, CD105+, and CD62E+ are all elevated in women with preeclampsia, and the first two are associated with cell apoptosis. They have also been shown to have a positive correlation with the sFlt1 : PIGF ratio, suggesting that the antiangiogenesis is related to apoptosis of the endothelial cells. Functionally, González-Quintero and colleagues have shown in women with preeclampsia that levels of CD31+/42 and CD62E+ EMP were significantly correlated with mean arterial pressure and worsening levels of proteinuria [61]. Others have reported delayed clearance of these EMPs up to 1 week afterartum, supporting the theory that endothelial damage persists in women with preeclampsia and may be related to its long-term sequelae [58]. 

### 6.4. The Role of Inflammation

Though the exact components which elicit the release of microparticles in response to endothelial injury in a preeclamptic woman are unknown, some have implicated proinflammatory cytokines, since these are known to be endothelial activators. Cytokines, such as tumor necrosis factor-*α* (TNF-*α*) and IL-1, have been shown to be elevated in preeclampsia [62]. The role of TNF-*α* in mediating the increase in SFlt-1 in response to placental ischemia was recently discussed [63]. Freeman and colleagues have recently confirmed these observations and additionally reported the significant elevation of IL-6/IL-10 ratio in women who had had preeclampsia twenty years earlier, showing the persistence of the inflammation changes [64]. In comparing the gene expression in placental tissue from preeclamptic women with tissue from those with a strong family history of heart disease, genes relevant to immune function and inflammatory responses have been shown to be similarly expressed in these 2 conditions [65]. Because of these findings, preeclampsia has been characterized as an intense systemic inflammatory environment [66]. Since women suffering from autoimmune conditions are known to have an increased risk of developing the syndrome, one of the triggers to widespread endothelial dysfunction may indeed be the immune and inflammatory modulations that occur in preeclampsia. Interestingly, neonates born following preeclamptic pregnancies also show elevated levels of IL-8 and natural killer (NK) cells with reduced T cell function [67–70].

### 6.5. Epigenetic Modulation

Our understanding of how reduced uterine perfusion and hypoxia promote the release of inflammatory cytokines and derange antiangiogenic factors leading to systemic endothelial and vascular dysfunction is still limited. A proposed hypothesis supporting the inheritability of these vascular changes involves epigenetic programming, whereby the polymorphisms for certain genes, such as those encoding eNOS or inflammatory modulators, are activated by adverse exposures including early placental insufficiency or hypoxia. This activation may then lead to changes in transcriptional function of downstream cytokines or antiangiogenic proteins. The level of endothelial damage can now be quantified through EMPs which may serve as a biomarker for both present and future cardiovascular risks [45, 61].

## 7. Mitigating the Blind Spot in the Healthcare System

The health consequences of preeclampsia to the mother are so clear that a recent review has suggested that this condition may be a better predictor of future vascular risk in the mother than pregnancy-associated hypertension or diabetes [26]. This review additionally emphasizes that significant health consequences occur in those born to preeclamptic mothers. Fortunately, there are now significant measures that can be taken to reduce the risk of vascular events if an individual of any age is known to be at increased risk for vascular disease in organs such as the brain and the heart [71]. These measures may include proactive observation of blood pressure, and if needed treatment of hypertension, and lifestyle changes that may mitigate this and other complications. For example, the recent literature shows that a higher level of total daily physical activity protects against cognitive decline [72] and brain atrophy [73]. We are unaware, however, of any systematic effort aimed at informing the progeny of preeclamptic mothers that they have increased risk for vascular complications and proactively offering them close follow-up. It is our observation that patients with vascular disease are usually unaware of the health status of their mothers during the pregnancy and at the time of their delivery. This information should be made available to them, but this must be done within accepted ethical boundaries, including the prior consent of the mother to make this information available. This also assumes that the individual whose mother was preeclamptic wishes to know this information. Once these boundaries are respected and verified, the advent of electronic medical records may facilitate this task, although this is not universally available. In the meantime, it is our recommendation that pediatricians, family physicians, and other health practitioners should suggest to those in their care to seek out this information and make it part of their permanent health record. Once known as a product of a preeclamptic pregnancy, the individual should be advised to monitor vascular risk factors and to remain in contact with the health care providers to benefit from preventive measures. As this review illustrates, the consequences of not doing so can be significant.
